# Physicochemical Properties and Atomic-Scale Interactions in Polyaniline (Emeraldine Base)/Starch Bio-Based Composites: Experimental and Computational Investigations

**DOI:** 10.3390/polym14081505

**Published:** 2022-04-07

**Authors:** Soufiane Boudjelida, Souad Djellali, Hana Ferkous, Yacine Benguerba, Imane Chikouche, Mauro Carraro

**Affiliations:** 1 Laboratory LMSE, University Mohamed El Bachir El Ibrahimi, Bordj Bou Arreridj 34030, Algeria; soufianeboudj@live.fr; 2 Department of Chemical Sciences, University of Padova, Via Marzolo 1, 35131 Padova, Italy; 3 Laboratoire de Physico-Chimie des Hauts Polymères, University Ferhat Abbas Setif-1, Setif 19000, Algeria; 4 Department of Chemistry, Faculty of Sciences, University Ferhat Abbas Setif-1, Setif 19000, Algeria; 5 Laboratoire de Génie Mécanique et Matériaux, Faculté de Technologie, Université de 20 août 1955 de Skikda, Skikda 21000, Algeria; hanaferkous@gmail.com; 6 Département de Technologie, Université de 20 août 1955 de Skikda, Skikda 21000, Algeria; 7 Department of Process Engineering, Faculty of Technology, University Ferhat Abbas Setif 1, Setif 19000, Algeria; yacinebenguerba@univ-setif.dz; 8 Laboratoire Croissance et Caractérisation de Nouveaux Semi-Conducteurs, Faculté de Technologie, Université Sétif 1, Setif 19000, Algeria; chikouche_imene@yahoo.fr; 9 ITM-CNR, UoS of Padova, Via Marzolo 1, 35131 Padova, Italy

**Keywords:** biocomposites, polyaniline colloids, starch, steric stabilizer, cyclic voltammetry, DFT, AIM study, COSMO-RS

## Abstract

The processability of conductive polymers still represents a challenge. The use of potato starch as a steric stabilizer for the preparation of stable dispersions of polyaniline (emeraldine base, EB) is described in this paper. Biocomposites are obtained by oxidative polymerization of aniline in aqueous solutions containing different ratios of aniline and starch (% *w*/*w*). PANI-EB/Starch biocomposites are subjected to structural analysis (UV-Visible, RAMAN, ATR, XRD), thermal analysis (TGA, DSC), morphological analysis (SEM, Laser Granulometry), and electrochemical analysis using cyclic voltammetry. The samples were also tested for their solubility using various organic solvents. The results showed that, with respect to starch particles, PANI/starch biocomposites exhibit an overall decrease in particles size, which improves both their aqueous dispersion and solubility in organic solvents. Although X-ray diffraction and DSC analyses indicated a loss of crystallinity in biocomposites, the cyclic voltammetry tests revealed that all PANI-EB/Starch biocomposites possess improved redox exchange properties. Finally, the weak interactions at the atomic-level interactions between amylopectin–aniline and amylopectin–PANI were disclosed by the computational studies using DFT, COSMO-RS, and AIM methods.

## 1. Introduction

A variety of reports have emerged in recent decades documenting the use of sterically stabilized colloidal dispersions of conductive polymers, which are much more processable than traditional ones [1]. In the case of dispersion polymerization, and when the reaction medium contains a soluble stabilizer, the macroscopic aggregation and precipitation of the resulting conductive polymers can be avoided, leading to submicrometer well-dispersed particles [2]. In this context, several water-soluble polymers, such as cellulosic derivatives [3,4], poly(vinyl alcohol) [5], poly(ethylene oxide) [6], and poly(methyl vinyl ether) [7], were used as steric stabilizers to overcome the processing hardness of conductive polymers, which is related to their very low solubility in common solvents and/or infusibility, limiting their applications.

Polyaniline (PANI) is among the most studied and developed organic conductive polymers. Its large-scale applications stem from its good redox exchange properties, its simple and cheap preparation methods as well as its great environmental stability [8,9]. However, as with other conductive polymers, polyaniline is very difficult to be processed and the preparation of colloidal PANI-based dispersions seems to be a promising way to overcome such a problem [10]. Several workers have taken advantage of this approach to produce processable dispersions including PANI/poly(methyl methacrylate) particles stabilized in hydrocarbons [11], PANI stabilized with partially hydrolyzed poly(vinyl alcohol) [12] or with poly(N-vinylpyrrolidone) [13], and PANI/hydroxypropyl cellulose [14]. 

Numerous studies were also conducted to exploit biopolymers issued from renewable natural and agricultural resources, to develop novel bio-composites with conductive polymers, thus leading to entirely new directions in the research of advanced functional materials for a sustainable future [14,15]. In such a case, the water-soluble or water-dispersible biopolymers bind PANI chains and lead to stabilized colloids [16,17,18]. In this context, the “intelligent behavior” of PANI colloids prepared with chitosan or poly(N-isopropyl acrylamide) [19,20], cellulose derivatives [21], nanocellulose [22] and pectin [23,24], as steric stabilizers, was reported.

The resulting biocomposites could not only improve the processability of PANI, but also enhance its biocompatibility [25,26,27]. For example, PANI-based materials have been used for the detection and analysis of bio-molecules (enzymes, antibodies, DNA, proteins, etc.), making them suitable candidates for biomedical applications [25,27,28]. On the other hand, even if in vivo studies have shown that the emeraldine base form of PANI with electroactive properties [29] did not provoke inflammatory responses in a rodent model, suggesting good tolerance and bio-/histo-compatibility [30], risks may arise from the presence of polymerization by-products (benzidine, aniline dimers, and oligomers). Such by-products, indeed, can be cytotoxic, also with carcinogenic effects. All in all, although their presence can be avoided by suitable washing steps [31], the use of PANI-based materials still deserves many precautions. 

In our work, we have used starch, which is the most abundant polysaccharide produced by higher plants, and it is extracted intracellularly in the form of granules of 2–100 µm in diameter [32]. In general, starches are semi-crystalline polymers made of a mixture of amylose and amylopectin chains, with a crystallinity of about 20–45% [33]. 

Starch was used by several authors to develop stabilized latexes [34,35,36]. In the work of Pradeep et al. [34], in particular, flexible semiconducting thin films from pure vulcanized natural rubber latex with enhanced electrical conductivity were produced. Other starch-stabilized colloids were also developed with bimetallic nanoparticles to improve the stability and reactivity of nanoparticles [37,38,39], PLGA (polylactic-co-glycolic acid) nanospheres [40], and PMMA (polymethylmethacrylate) lattices [41]. 

The usage of starch as a non-toxic and biodegradable biopolymer to elaborate PANI-based composites has led to the formation of advanced functional materials for various applications (water remediation, energy generation, and storage, electrochemical, medical and biomedical applications, etc.) [15,42,43,44,45,46]. In the same framework, we have attempted to elaborate water dispersible and processable polyaniline using starch as a steric stabilizer. The oxidative chemical polymerization of polyaniline was performed in an aqueous solution containing various ratios of aniline to starch (*w*/*w*). The obtained PANI/starch biocomposites were characterized by spectroscopic, microscopic, thermal, and electrochemical techniques, in order to assess the most promising PANI: starch ratio. Moreover, computational calculations based on DFT were carried out to find missing information about the interactions between starch–aniline and starch–polyaniline during the synthesis and in the final form of the biocomposites. 

## 2. Materials and Methods

### 2.1. Synthesis of PANI/Starch Biocomposites

Polyaniline/starch materials (Table 1) were prepared by in situ polymerization of aniline in the presence of aqueous starch solutions under magnetic stirring. In a typical procedure [47], in a three-necked flask, aniline was added to the aqueous dispersions of starch at various ratios of aniline: starch (*w*/*w*) at room temperature (20 °C) and stirred for 4 h. The chemical polymerization of aniline was carried out at low temperatures in an ice bath (T~0–4 °C) using an APS (ammonium persulfate) oxidative solution. The mixture was maintained under magnetic stirring for 3 h, then left without stirring for 48 h to allow the majority of aniline to react, after which the dark powder obtained was filtered, washed with water and acetone to remove low-molecular-weight reaction by-products, i.e., aniline dimers and oligomers, then dried at 40 °C for 48 h.

### 2.2. Characterization 

Structural characterization of samples was carried out by FTIR spectroscopy using a Nicolet 5700 spectrometer (Thermo Fisher, Waltham, MA, USA), equipped with a diamond ATR sampling accessory, on the powdered material, UV-Visible spectroscopy using a Cary 5000 UV−vis−NIR spectrometer (Varian, Palo Alto, CA, USA) on solutions prepared by dissolving 2 mg in 10 mL of DMF, and RAMAN spectroscopy using a DXR2 Raman spectrometer (Thermo Scientific, Madison, WI, USA), operating at 532 nm. The solubility tests were performed at different temperatures (20, 40, 50, 60, 80 °C) by dispersing the same weight of all materials in 10 mL of solvents, to obtain a dilute concentration (0.2% *w*/*v*). The solvents used are water, acetone, methanol, ethanol, glycerol, ethyl acetate, chloroform, toluene, hexane, cyclohexane, DMSO, and DMF. While colorless supernatants were obtained with insoluble mixtures, the presence of residual solids, in the case of partially soluble mixtures, was observed by a lens.

Homopolymers and biocomposites were subjected to laser granulometry analysis by using a CILAS 1190 analyzer (CPS Us, Inc., Madison, WI, USA). Thermal gravimetric analyses (with a TGA Q5000 instrument, TA Instruments, Hüllhorst, Germany) were performed until 700 °C (10 °C/min), while DSC (with a TA instrument Q20) was set from room temperature to 300 °C at 10 °C/min, under a nitrogen atmosphere; thermodynamic parameters were calculated by using the software TA Universal Analysis. X-ray diffraction analysis was performed using CuKα radiation (λ = 1.54 Å) (X’Pert3 Powder apparatus) from 2θ = 10° to 60°, at a scan rate of 0.02 s^−1^; the peak crystalline areas were determined by using the X’pert HighScore software. SEM images of the composites were obtained after metallization with sputter quorum Q 150R E settings using Quanta 200 apparatus (FEI, Hillsboro, OR, USA). Cyclic voltammetry tests (with PGZ 301 potentiometer, Radiometer Analytical SAS, Lyon, France) were examined in a three-electrode system constituted of a reference electrode (Ag/AgCl), an auxiliary platinum electrode, and a carbon electrode as the working electrode. The electrolyte was a KCl solution (1 M) and the voltammograms were recorded in a voltage window between −0.8 and +0.8 mV at various scan rates (10, 50, and 100 mV s^−1^).

## 3. Computational Study

Geometric optimization of the molecules was performed with the Dmol3 module [48] utilizing a modeling package created by Accelrys Incorporation (Cambridge, UK): Materials Studio 2017TM [49], B3LYP functional [50], and DNP basis set (4.4 basis file) [51].

The HOMO (Highest Occupied Molecular Orbital) and LUMO (Lowest Unoccupied Molecular Orbital) frontier molecular orbitals (FMOs) were investigated [52]. The high value of the energy ξ_HOMO_ justifies the inclination to transfer electrons to a suitable acceptor molecule. In contrast, the low value of the energy ξ_LUMO_ justifies the molecule’s capacity to accept electrons from donor molecules [53].

The electronic chemical potential (μ) and global hardness (η) were determined using the ξ_HOMO_ and ξ_LUMO_ energies [54,55,56]:

µ = (ξ_HOMO_ + ξ_LUMO_)/2 (1)

ƞ = (ξ_LUMO_ − ξ_HOMO_)/2 (2)

The chemical potential µ is related to the electrophilicity index ω by the following relationship [57]:

ω = (µ^2^)/(2 ƞ) (3)

ω indicates an electrophile’s capacity to gain an extra electrical charge [58].

The optimal fraction of transferred electrons (ΔN) is given by [59]:

ΔN = −µ/ƞ(4)

COSMO-RS (Conductor-like Screening Model for Real Solvents) is a thermodynamic-based quantum chemistry technique for determining chemical potentials in solutions [60]. For each species in the solution, this technique can estimate sigma charge densities and chemical potentials. The computation is divided into two parts: first, the molecule was geometrically optimized; then, using the COSMOtherm program [61] (p. 2), sigma profiles and sigma potentials were computed using the acquired cosmo-files.

Intermolecular interactions may be calculated using the Atoms in Molecule (AIM) method. The main goal of AIM is to examine the character and strength of the bonding interaction in molecular systems by using the electron density ρ(r) of the molecules as a tool. Based on the second derivative, ∇^2 ρ(r) sign [62,63], the kind of chemical bonds may be identified at the bond critical point (BCP) where it is minimum. As a result, if the ρ value is large and ∇^2 ρ(r) is negative, the bond is covalent (polar). On the other hand, positive ∇^2 ρ(r) indicates that the kinetic energy G(r) is greater than the potential energy V(r) [64]. The |V|/G ratio indicates that the system’s ability to group electrons, V(r), and its ability to dilute them through electronic mobility, G(r), compete within the system. As a result, the ratio |V|/G > 1 indicates that an excess of electric charges is utilized to generate the interaction, whereas |V|/G < 1 indicates the opposite. The total energy density, H(r) = V(r) + G(r), in the first situation, has a negative value, whereas in the second case, it has a positive value. Thus, the categorization of interactions is based on the values of that indicator: (i) pure interactions with closed layers, |V|/G < 1; (ii) interactions with closed layers, 1 < |V|/G < 2; and (iii) interactions with the shared layer, |V|/G > 2. The first two forms of interaction are ascribed to hydrogen bonding, whereas the third type is a covalent interaction. Furthermore, positive, ∇^2 ρ(r), and H values indicate electrostatic contact, whereas negative values indicate a covalent bond. A partly covalent bond is defined as a positive ∇^2 ρ(r) combined with a negative H value [65].

The Amsterdam Density Functional (ADF) program [66,67] was used to perform AIM calculations. For molecular structure optimization, the DFT method was employed. The AIM research [67,68] used the def-TZVP basis set to describe exchange correlation effects between these variables (Becke 3-Parameter, Lee, Yang, and Parr).

## 4. Results and Discussion

### 4.1. Physico-Chemical Properties

**Figure 1:**
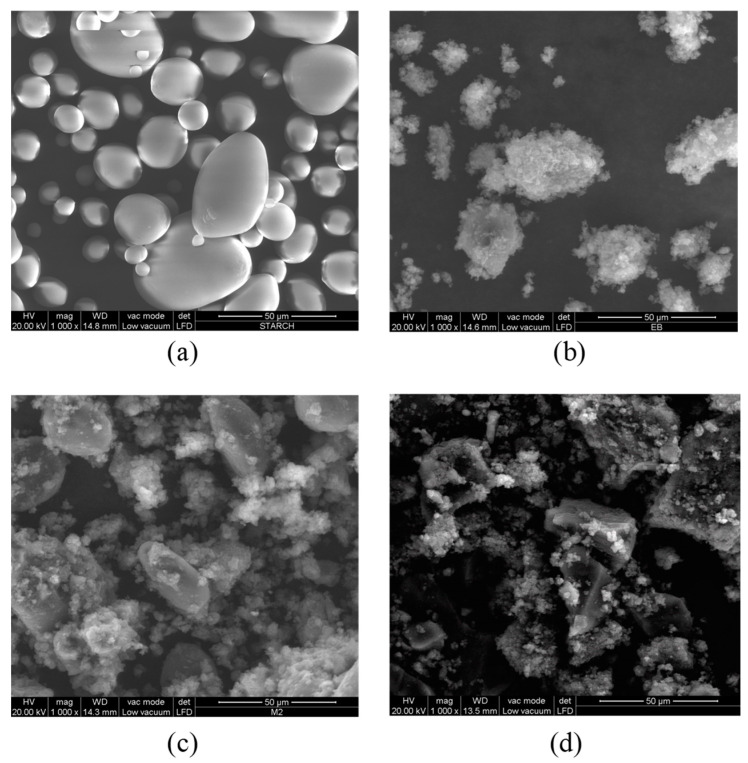
SEM images of (**a**): starch, (**b**) PANI and PANI/starch biocomposites. (**c**): (1:1) and (**d**): (3:1).

The scanning electron micrographs for polyaniline, starch, and polyaniline/starch biocomposites are shown in Figure 1a–d. The SEM micrograph of starch in Figure 1a reveals pseudo-spherical granules with smooth and well-defined surfaces. Figure 1b–d represent SEM images of starch/polyaniline composites with different ratios of aniline to starch. The figure shows that, in the case of a composite, when aniline is polymerized in the presence of starch, the polyaniline chains produced are grafted or adsorbed on this steric stabilizer. At the lowest concentration of aniline, the surface of the starch granules is rougher than that of pure starch, suggesting that aniline polymerization occurs on the surface of the granules of starch, forming an irregular coating over it [42,47]. However, as the concentration of aniline increases, more of the starch surface is covered and an overgrowth of polyaniline is observed over the starch particles [45]. 

From the analysis of particle size by laser granulometry, it can be seen that the particles of the obtained PANI/starch materials exhibit a wide distribution of particle sizes revealing a high polydispersity of particles (relative mean variance of the particle size distribution), which may indicate the occurrence of some aggregation of the biocomposites particles [69]. This statement is consistent with the findings of other authors [70,71]. Furthermore, as shown in Table 2, homopolymers and biocomposites particles exhibit average diameters ranging from 28.8 µm to 68.9 µm. It is also worth noting that all composites possess particle sizes lower than that of polyaniline, whereby the composite with the highest aniline content exhibits the lowest average diameter. Thus, the growing polyaniline chains may affect the intramolecular forces along the polymer chains of amylose and amylopectin, leading to smaller particles [15].

The capacity of the various solvents (water, acetone, methanol, ethanol, glycerol, ethyl acetate, chloroform, toluene, hexane, cyclohexane, DMSO, and DMF) to dissolve the prepared materials was examined at different temperatures. The results obtained (Table 3) revealed that PANI-EB, starch, and PANI-EB/starch composites are insoluble in the majority of the solvents used even at high temperatures (80 °C). However, starch is soluble in DMSO but slightly soluble in water and DMF, whereas PANI-EB is slightly soluble in DMSO and soluble in DMF, and it is well known that it is not soluble in most common solvents [72]. The solubility behavior of PANI-EB/starch biocomposites differed from that of homopolymers where we noticed a good solubility in DMF and DMSO and an enhanced dispersion in water at 20–40 °C before more homogeneous mixtures were achieved at higher temperatures (60–80 °C). A further enhancement in the solubility was observed with glycerol and chloroform mainly at higher temperatures (>40 °C) with good dispersions at low temperatures. These findings indicate that the surfaces of the products have, at different temperatures, different affinities for each solvent. Nevertheless, the remaining solvents were not able to dissolve or even disperse PANI/starch biocomposites.

### 4.2. Structural Characterization 

**Figure 2:**
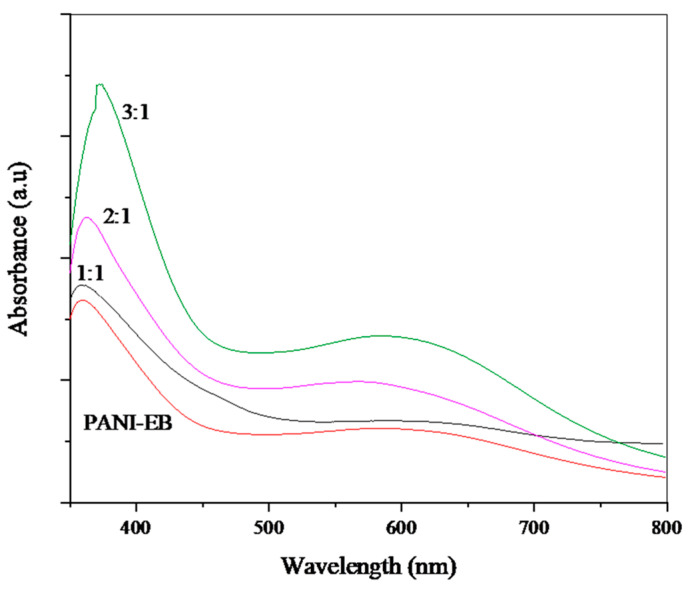
UV-vis spectra of PANI, starch, and PANI/starch biocomposites in DMF.

**Figure 3:**
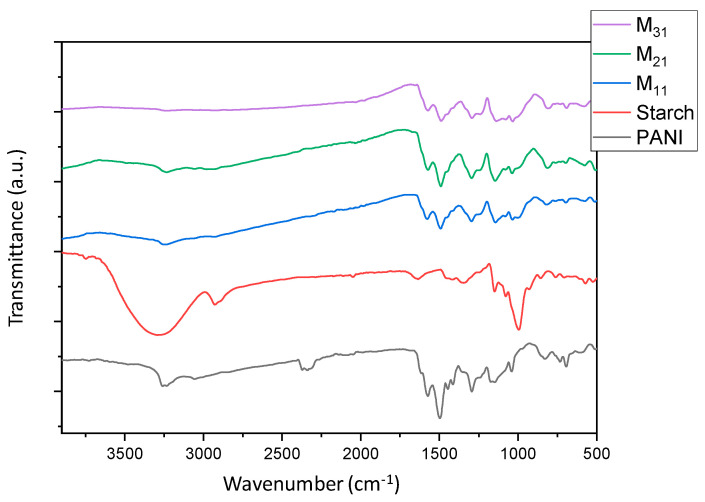
FTIR-ATR spectra of PANI, starch, and PANI/starch biocomposites. The residual CO_2_ signal may be due to the adsorption properties of PANI.

**Figure 4:**
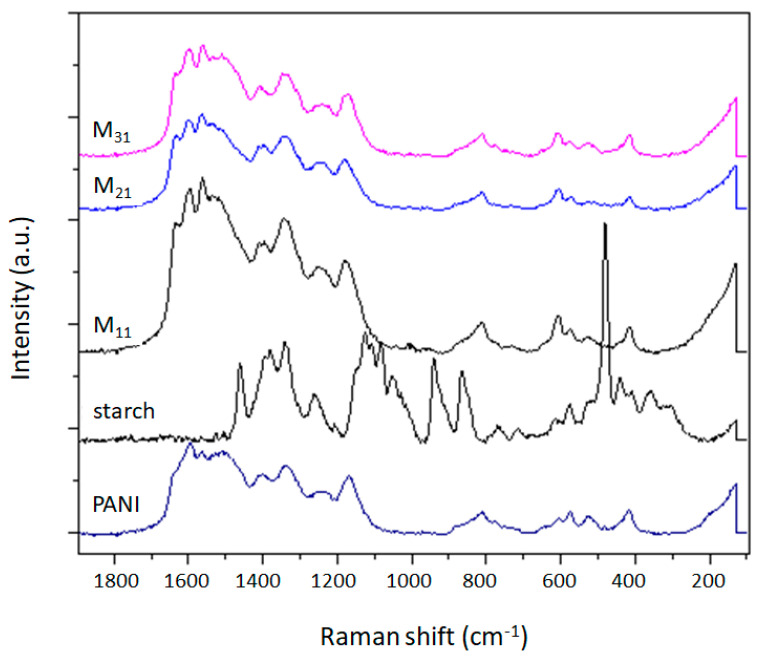
RAMAN spectra of PANI, starch, and PANI/starch biocomposites.

The UV-visible, FTIR, and RAMAN spectra of our products are shown in Figure 2, Figure 3 and Figure 4, respectively. The UV-visible absorption spectra in Figure 2 display two broad absorption bands around 315–360 nm and 560–600 nm for all composites, which confirm the occurrence of the PANI-EB backbone structure. The first peak is assigned to π–π* transition occurring from the highest occupied molecular orbital to the lowest unoccupied molecular orbital of the amine benzenoid rings, while the second peak is ascribed to benzenoid–quinoid rings’ charge transfer [73]. The difference in the relative intensities of the peaks observed can be due to the variation in the quantities of benzenoid and quinoid units present in the materials as the ratio of aniline to starch varies. Moreover, the red shift for the 3:1 sample may be due to a higher amount of longer chains as well as to a growing contribution of a less oxidized form of PANI [74,75]. 

Figure 3 shows the FTIR-ATR spectra of polyaniline, starch, and their biocomposites. For the native starch, the broadband characteristic for the O-H groups of starch appears at 3252 cm^−1^ and the peak around 2924 cm^−1^ is attributed to an asymmetric stretching vibration of the C-H bond in pyranoid rings. Several other adsorption bands between 854 cm^−1^ and 1415 cm^−1^ are attributed to the contribution of various functional groups such as C-O and C-O-C [47]. Moreover, the peaks around 991 to 994 cm^−1^ are ascribed to the “C-O” bonds resulting from “C-O-H” and those of the anhydrous glucose rings in the starch backbone [76]. The characteristic peaks of pure PANI have been reported in previous works [42,47]. The bands appearing at 1539 cm^−1^ and 1456 cm^−1^ can be assigned to the vibration of quinoid rings (“N=Q=N”) and benzenoid rings (“N-B-N”), respectively. The bands at 1282 cm^−1^ and 1230 cm^−1^ correspond to the “C-H” stretching vibration within the aromatic conjugation. The presence of PANI is confirmed in all composites’ spectra in which PANI and starch are present at different concentrations. As PANI is more exposed on the surface of the particles, its signals dominate the spectra; moreover, the strong interaction between the chains may be responsible for the disappearance of some typical starch bands [77]. 

The Raman spectra of PANI, starch, and their biocomposites, obtained using an exciting wavelength of 532 nm, are displayed in Figure 4. This method is very efficient for understanding the interaction among various components and provides information about the chemical structure of different domains in the composite systems. 

From the spectrum of PANI, we can observe some characteristic bands rinsing from carbon–carbon, carbon–hydrogen, and carbon–nitrogen vibration modes. Benzenoid rings exhibited C-C stretching modes between 1520 cm^−1^ and 1650 cm^−1^ and C-H bending modes between 1100 cm^−1^ and 1210 cm^−1^; however, quinoid rings give rise to C=N, and C-C ring stretching vibrations, which appeared at 1382 cm^−1^ and 1595 cm^−1^, respectively. Further bands are observed in the region 1210–1520 cm^−1^ for C-N stretching modes (amines, imines) and in the region 1100–1140 cm^−1^ characteristic of the PANI backbone. A weak band at 1170 cm^−1^ has been assigned for in-plane deformation of the C-C bond of the quinoid ring of PANI [46,78]. 

The observed bands in the Raman spectrum of starch, which are related to C-H and CH_2_ vibrations (deformation, bending), appear in the region 1460–1340 cm^−1^ and at 862 cm^−1^. The signals related to C-O-H bending vibrations arise at the wavenumbers 1335, 1123, and 1080 cm^−1^. At 1257 cm^−1^, the band related to vibrational modes of CH_2_OH appears, while those associated with the skeletal mode involving α-(1–4) linkage appear at 942 cm^−1^ and between 770–360 cm^−1^ [79]. 

Similar to FTIR-ATR, the Raman curves of PANI/starch biocomposites display the typical peaks of PANI (Figure 4), with no meaningful shift. Moreover, knowing that Raman spectroscopy is a surface thickness-dependent tool [80,81], the response of PANI/starch biocomposites to laser excitation could be taken as a piece of further evidence that starch grains are covered by a thick PANI chain layer, which prevents the penetration of the laser beam to starch macromolecules and consequently impedes the appearance of starch characteristic peaks. These results concord with the SEM images in Figure 1. 

### 4.3. X-ray Diffraction Pattern (XRD)

**Figure 5:**
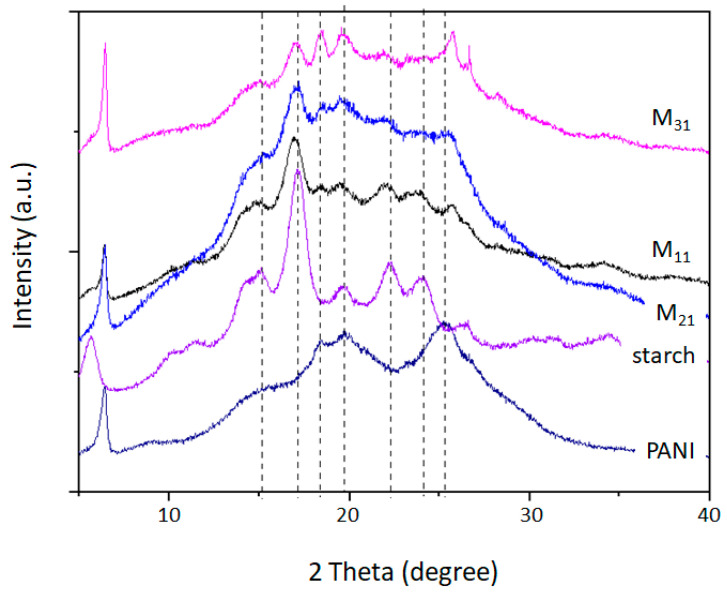
XRD diffractograms of starch, PANI, and PANI/starch biocomposites.

The X-ray diffraction patterns of starch, polyaniline, and PANI/starch biocomposites are presented in Figure 5. A semi-crystalline pattern can be observed for potato starch that exhibits strong reflection at 2θ = 17.2°, moderate reflections at 2θ = 5.7°, 15.04°, 22.22°, and 24.1°, in addition to weak reflections at 2θ = 15.2° and 19.6°. Such a pattern is typical for B-type crystalline structure starches (Tuber starches) [82,83]. The XRD profile of PANI-EB representing the crystalline parts shows many broad reflection peaks, located at 2θ = 6.4°, 9.1°, 15.7°, 18.4°, 19.8°, and 25.4°. Larger peaks are believed to be formed by many reflections of wider clusters [84]. 

The interplanar distance ‘d’ is calculated for each peak using Bragg’s equation:

nλ = 2d sinθ (5)

where λ = 1.542 Å, n = 1 (first-order reflection), θ = the Bragg angle for the corresponding peak.

The diffraction spacing and Miller indices (hkl) for our materials are displayed in Table 4. 

The values of the degree of crystallinity (Xc%) were obtained as a ratio between the peaks crystalline area and the total area of the XRD pattern under the curve. The following equation is employed [85]:

Xc (%) = Hc/(Hc + Ha) × 100(6)

where Hc and Ha are the intensities for the crystalline and amorphous profiles.

The crystallinity degree values of polyaniline and starch, as well as of biocomposites are summarized in Table 5. The native starch shows a high crystallinity (37%) when compared with polyaniline (19%). For PANI–starch biocomposites, a contribution due to different starch content can be confirmed. Changes in XRD patterns could be noticed, which may reveal alterations in the ultrastructure of the microdomains of PANI and starch mainly due to changes in the crystallinity. Such an observation is supported by the disappearance of starch peaks at 2θ = 22.22° and 26.58° and all PANI-EB peaks. Thus, it appears that the existence of aniline in the reaction medium and the PANI chains in propagation, partially destroy the original crystalline lattice of starch structure, and this is particularly evident for M_21_ and M_31_. On the other hand, it seems that polyaniline chains can form an organized structure, which is also reflected in the diffractograms, although with a lack of peaks attributed to the PANI-EB crystalline structure.

### 4.4. Thermal Characterization 

**Figure 6:**
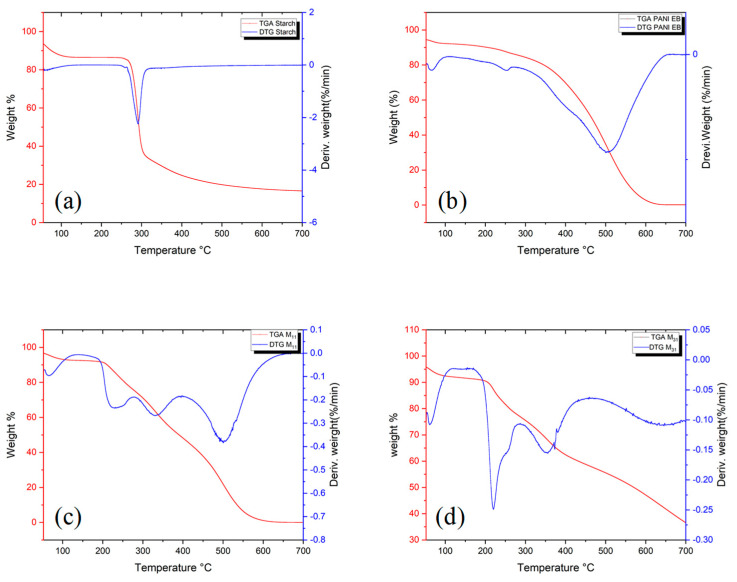
TGA and first derivatives curves of, (**a**): starch, (**b**): PANI EB and their biocomposites (**c**): M_11_, (**d**): M_31_.

The thermal behaviors of PANI, starch, and PANI/starch biocomposites were studied, under nitrogen, by TGA and DSC analyses. The corresponding thermogravimetry/differential thermogravimetry (TG/DTG) curves, depicted in Figure 6a–e, point out a weight loss within a broad range for polyaniline and as a sharp step for the degradation of starch. PANI-EB exhibits the first weight loss (Figure 6a) between 40 °C and 100 °C, which could be due to the evaporation of water and residual volatile molecules, followed by a small endothermic peak observed between 210 °C and 250 °C. A massive weight loss starts from 400 °C, owing to the degradation of the polyaniline chains [86]. The native starch undergoes 50% weight loss at 290 °C, while for polyaniline, a corresponding weight loss is noticed at 461 °C (Table 6). Furthermore, the DTG curves also show an overall decrease in the degradation rate for biocomposites with the rise in aniline amount, indicating a strong influence of polyaniline on the thermal stability of starch. As a matter of fact, the typical decomposition feature of PANI, with a strong DTG peak at 500 °C, is only present for M11, while M21 (not shown) and M31 show a quite regular weight loss till the limit of the explored range.

Detailed data can be derived from the TGA thermograms, which are related to the thermal stability such as T5, T50, and T95, defined as the temperature at which 5, 50 and 95% of the mass is volatilized, respectively. As observed in Table 6, PANI-EB and biocomposites are remarkably more stable than starch. 

**Figure 7:**
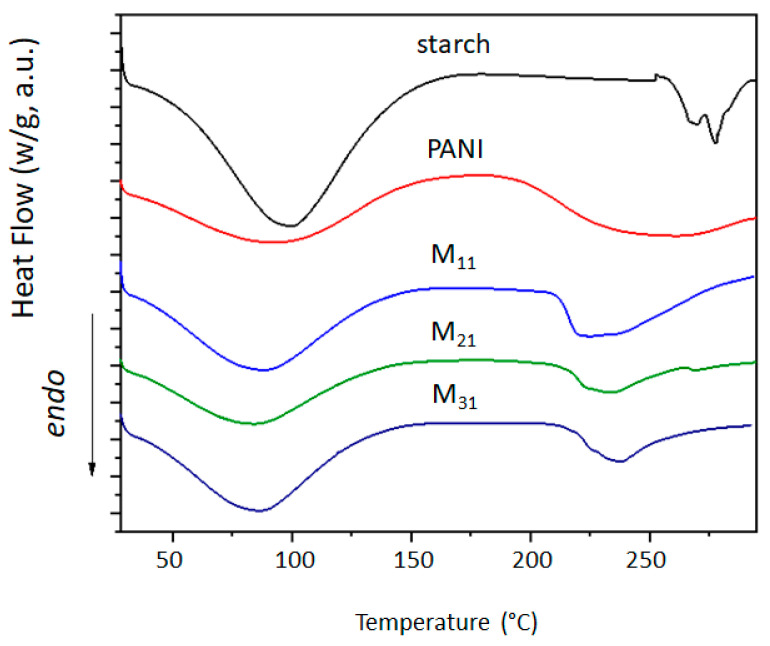
DSC thermograms of starch, PANI, and PANI–starch biocomposites.

Figure 7 displays the DSC profiles of polyaniline, starch, and their biocomposites. Broad endothermic peaks are perceived in the temperature range of 60–120 °C, which may correspond to the evaporation of water as supported by the TGA and DTG data. Moreover, the PANI thermogram shows another endothermic step starting from 200 °C, which could be attributed to the glass transition temperature (Tg), where the movement of small chain segments starts [87,88]. On the other hand, the thermogram of starch reveals two narrow endothermic peaks centered at 266 °C and 278 °C that are attributed to the thermal decomposition of starch chains [89]. This result corroborates with the TGA obtained data. Regarding PANI/starch materials, we observe the presence of the endothermic peak related to water evaporation in the same temperature range, but the second endothermic peak, related to the variation in the Tg of polyaniline, shifts to a lower temperature, especially for M_11_, indicating structural modification in the biocomposites owing to the interactions between components. The results of DSC measurement are reported in Table 7.

### 4.5. Cyclic Voltammetry

**Figure 8:**
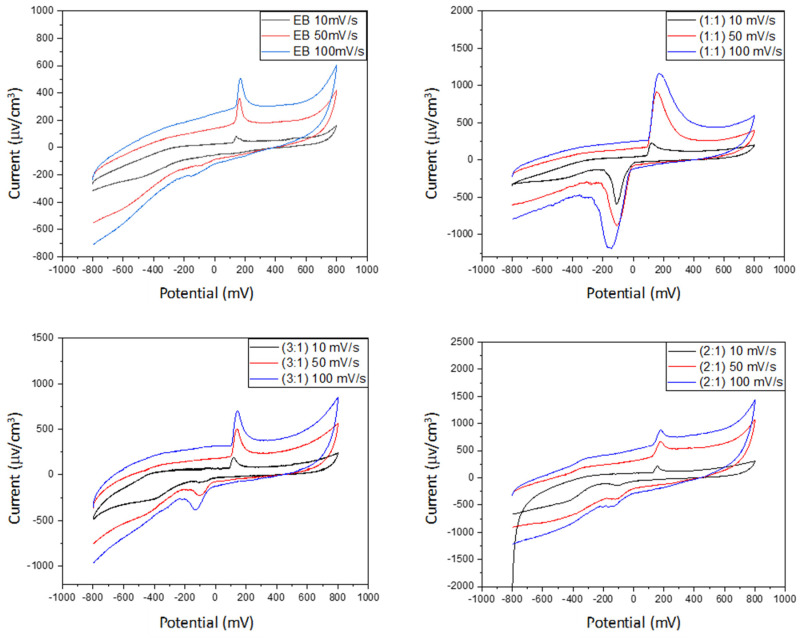
Cyclic voltammograms of PANI and PANI/starch biocomposites.

The electroactivity of PANI and PANI-EB/starch composites was studied in the supporting aqueous electrolyte (KCl) by cyclic voltammetry, and the recorded voltammograms are shown in Figure 8. All the curves displayed a pair of oxidation and reduction peaks, arising from the redox behavior of PANI-EB. Indeed, the peaks appearing for each oxidation and reduction process for PANI and PANI-based composites can be assigned to leucoemeraldine/emeraldine transition states of polyaniline [44,90]. In most cases, voltammograms exhibit a well-defined oxidation peak at about 120 mV that appears to be associated with a less-evident reduction peak at about 110 mV, highlighting the occurrence of a redox electron transfer [91]. 

The species mobility within the composite was evaluated from the dependence of the redox peaks intensity on the scan rate, from 10 to 100 mV/s. As reported in Table 8, the current density increases with the increasing scan rate value, and the waves show an increase in their areas with increasing scan rates, revealing the good electroactivity as well as the electrochemical capacitive behavior of our materials [44,92]. 

In order to better understand the electronic behavior of PANI and its composites with starch, we have plotted the variations in the current density of the oxidation peak for each sample as a function of the square root of the scan rate √v.

**Figure 9:**
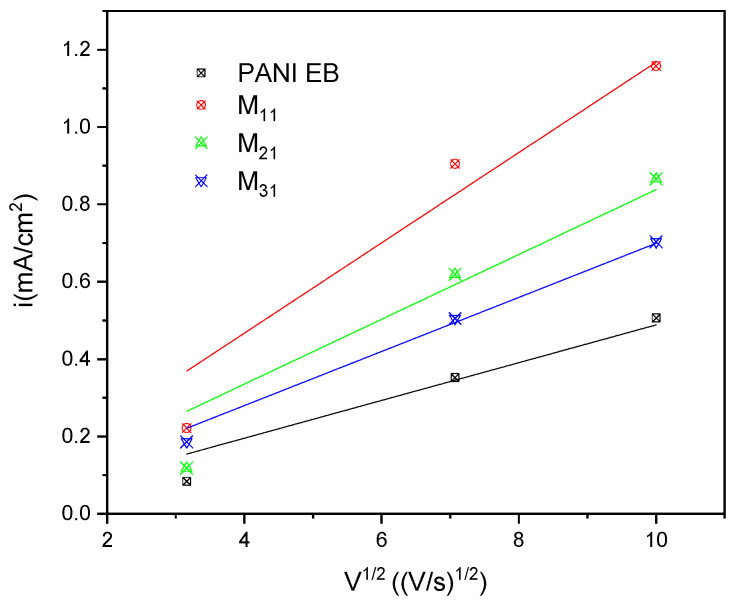
Peak current variation with scan rates.

We can observe in Figure 9 that the current density is linear with √v for all the synthesized materials. The oxidation phenomenon is therefore limited by the ion diffusion, and the transport of charged species to the electrode surface is ensured by this transport mode.

In fact, the Randles Sevcik relation gives a straight line when pure diffusion controls the electrochemical reaction.

I_pa_ = 2.69 × 10^5^ n^3/2^A D^1/2^ C v^1/2^ (7)

ip = current maximum in amps

n = number of electrons transferred in the redox event

A = electrode area in cm^2^

D = diffusion coefficient in cm^2^/s

C = concentration in mol/cm^3^

v = scan rate in V/s

Therefore, it is clear that the PANI synthesized by chemical oxidation polymerization is a conductor material. Note that according to our results, the M11 sample is the best conductor among these materials.

### 4.6. Computational Assessment

The main electronic properties calculated for the aniline, PANI, and starch are summarized in Table 9. As amylopectin is the most abundant component (85%) in the starch under investigation, it was considered a suitable model. According to the energy values, comparable values of the aniline isomers ξ_HOMO_ and ξ_LUMO_ indicate a quasi-equivalent tendency to donate or accept electrons. That said, isomers I1 and I2 have the highest values, showing that they are the more probable molecules participating in the polymerization process to produce the PANI chain (this is confirmed by the highest electron escaping tendency from an equilibrium system, μ = −3.027 eV). Both PANI and amylopectin are more reactive than the PANI parent species (isomers I1 or I2) (see Table 9). Regarding the gap values in Table 10, the amylopectin is the most stable in this set of polymers. 

In conclusion, PANI has the highest ability to acquire an extra electron charge (highest ω value: 8.386 eV) and the lowest resistance to charge transfer (the lowest hardness η value, 0.895) (LUMO of PANI, see Table 10). It is thus concluded that amylopectin is the electron donor with the highest ξ_HOMO_ = −7.056 eV (see Table 2). The results for ΔN are 4.328 electrons (acquired) for PANI and 0.894 electrons (released) for amylopectin.

The Fukui results shown Table 11 led to the same conclusion as HOMO and LUMO. The PANI’s f+ (LUMO) sites are localized on the N (72) (f+ = 0.076) and N (83) (f+ = 0.074) while the amylopectin’s f- (HOMO) sites are localized on the O (1) (f− = 0.105) and O (9) (f− = 0.115).

The sigma profile above is divided into three distinct regions:

- HBD Region—Hydrogen bond donor region: the sigma values are less than −0.01 eÅ^−2^. The negative sigma values mean positive polarities.
- Non-Polar Region—σ values are given in the interval −0.01 eÅ^−2^ to +0.01 eÅ^−2^.
- HBA Region—Hydrogen bond acceptor region: the σ values are greater than 0.01 eÅ^−2^. Positive sigma values represent negative polarities.

**Figure 10:**
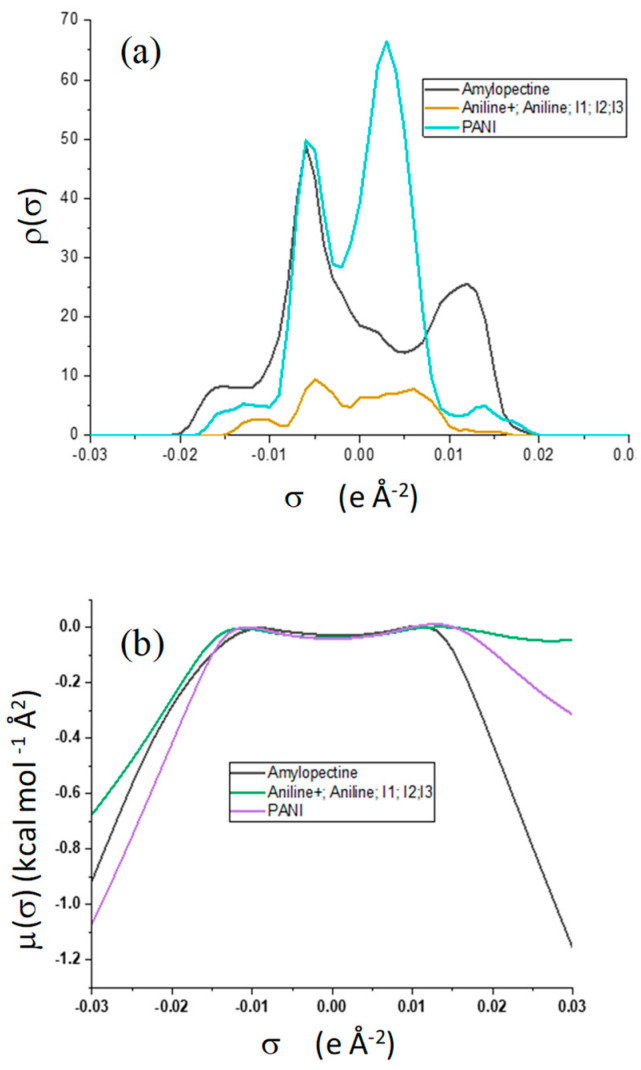
σ-profile (**a**) and σ-potential (**b**) distributions of aniline, polyaniline, and amylopectin.

Figure 10a shows that the highest peaks for all the selected molecules are in the non-polar region. They are showing the tremendous non-polar character of the molecule surfaces. Amylopectin has a net peak in the HBA region, while PANI is in the HBD region. In addition, PANI has the highest affinity to HBA, and amylopectin has the highest affinity to HBD (Figure 10b). This confirms the frontier molecular orbital study results.

The interaction energies reported in Table 12, reveal that the HB type contributes the most to the mixture’s energy (−31.33496 and −40.29382 Ha). The PANI–amylopectin pair (ratio 1:1) achieves the highest value, while I2-amylopectin (ratio 3:1) has the lowest. The second contribution to the mixture’s energy is Van der Waals (between −26.70916 and −27.83387 Ha), with the minimum value for the PANI–amylopectin couple (1:1) and the maximum value for I2-amylopectin (3:1). The mixture’s energy ranges from −55.25390 for I2-amylopectin (3:1) to −63.51928 Ha for PANI–amylopectin (Emisfit).

**Table 12:** Interaction energies of amylopectin with the various compounds, which are depicted in Figure 11.

Mixture	Mass Ratio	E_VdW_	E_Misfit_	E_HB_	E_mix_
Aniline + Amylopectin	1:1	−27.27875	8.19827	−37.56368	−61.26681
	2:1	−27.60525	8.45979	−34.01977	−57.78789
	3:1	−27.74166	8.61834	−31.70186	−55.44783
Aniline_i2 Amylopectin	1:1	−27.34835	8.15238	−37.28638	−61.10501
	2:1	−27.69050	8.39477	−33.66549	−57.58388
	3:1	−27.83387	8.53758	−31.33496	−55.25390
PANI Amylopectin	1:1	−26.70916	8.10636	−40.29382	−63.51928
	2:1	−26.94796	8.36426	−38.53551	−61.74186
	3:1	−27.02828	8.50672	−37.60637	−60.75059
	3:1	−27.02828	8.50672	−37.60637	−60.75059

Energies are given in Hartree.

**Figure 12:**
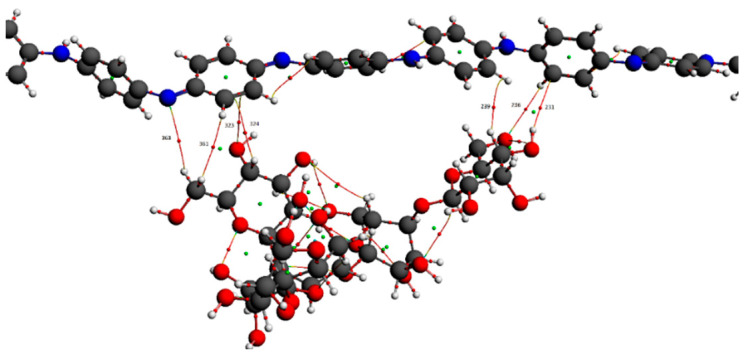
Molecular graph of the optimized PANI–amylopectin complex.

Figure 12 shows a molecular graph of the optimized PANI–amylopectin complex, while Table 13 and Table 14 give the topological characteristics at the BCP of interaction contacts in the PANI–amylopectin complex. The computed values of ρ(r) in BCPs are in the range [2.21 × 10^−3^–7.83 × 10^−3^] eV (Table 13), which are relatively low values, and ∇^2^2ρ(r) is positive, indicating the existence of hydrogen bond interactions in BCPs 231, 236, 239, 323, 324, 361, and 363 (BCPs are given in Table 13). The positive values of ∇^2^2ρ(r) and H demonstrate the H-bonding connection between the BCPs atoms (see Figure 12). Weak hydrogen bonding is characterized by E_HB_ < 0. The interaction between PANI and amylopectin is thus considered to be physical (with no covalent bonding).

## 5. Conclusions

The results of this work demonstrate a facile method for the synthesis of polyaniline colloids. In particular, potato starch was employed as a steric stabilizer to obtain stable dispersions of polyaniline (emeraldine base). Polyaniline/starch biocomposites with various aniline/starch ratios were thus synthesized via an oxidative polymerization and characterized. To evaluate the effect of aniline/starch ratios variation, several tests (UV-vis, FTIR-ATR, Raman, XRD, TGA, DSC), as well as computational calculation (DFT, COSMO-RS, AIM), were performed. This study demonstrates the possibility of using starch as a steric stabilizer, and so to empower the processability of the polyaniline by improving the solubility in DMSO and DMF and the dispersion behavior in water, as well as in glycerol and chloroform, at higher temperatures.

The growth of polyaniline is observed on the starch particles’ surfaces. The particle dimension and the degree of crystallinity appear to be strongly affected in the composite material, as demonstrated by the formation of smaller particles and the loss of the original starch crystalline lattice. Although less thermally stable, M11 appears to be the best in terms of morphology and retention of crystallinity degree. The cyclic voltammetry test shows better electro-activity in terms of the electron exchange and capacitive behavior of the composite materials, with the ratio of 1:1 being the best composition among these materials. Finally, the strong interactions between the PANI and amylopectin (the main starch component) were investigated by the DFT, COSMO-RS, and AIM methods, demonstrating their purely physical nature.

These results attest that the prepared composites as promising versatile materials, which are expected to broaden the electrochemical applications of PANI.

## Figures and Tables

**Figure 11:**
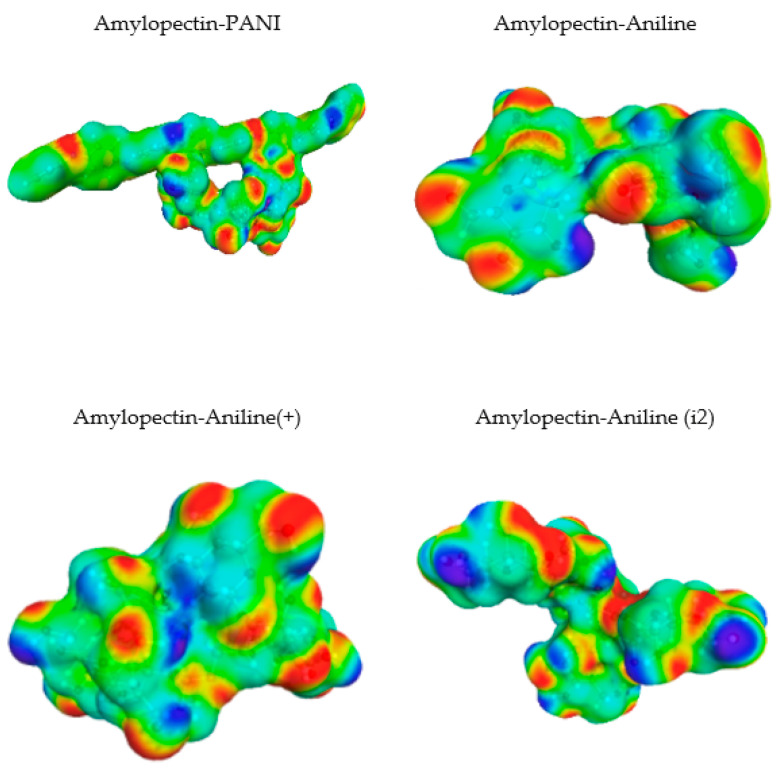
Interactions in PANI/starch biocomposites using COSMO-RS.

**Table 1:** Codes of the synthesized biocomposites.

Codes	M_11_	M_21_	M_31_
Aniline/Starch (*w*/*w*) ratio	1:1	2:1	3:1

**Table 2:** Average particle diameters of PANI, STARCH, and PANI/starch biocomposites.

	PANI EB	Starch	M_11_	M_21_	M_31_
Average diameter (µm)	68.9	38.9	40.2	31.5	28.8

**Table 3:** Solubility test of PANI, STARCH, and PANI/starch biocomposites.

T °C	Materials	Acetone	Methanol	Ethanol	Glycerol	Ethyl Acetate	Chloro-Form	Toluene	Hexane	Cyclohexane	DMSO	Water	DMF
20	M_11_	Δ	Δ	Δ	Δ	Δ	Δ	Δ	Δ	Δ	O	Δ	X
	M_21_	Δ	Δ	Δ	Δ	Δ	Δ	Δ	Δ	Δ	O	Δ	X
	M_31_	Δ	Δ	Δ	Δ	Δ	Δ	Δ	Δ	Δ	O	Δ	X
	PANI EB	Δ	Δ	Δ	Δ	Δ	Δ	Δ	Δ	Δ	O	Δ	X
	Starch	Δ	Δ	Δ	Δ	Δ	Δ	Δ	Δ	Δ	X	O	O
40	M_11_	Δ	Δ	Δ	Δ	Δ	Δ	Δ	Δ	Δ	X	Δ	X
	M_21_	Δ	Δ	Δ	Δ	Δ	Δ	Δ	Δ	Δ	X	Δ	X
	M_31_	Δ	Δ	Δ	Δ	Δ	Δ	Δ	Δ	Δ	X	Δ	X
	PANI EB	Δ	Δ	Δ	Δ	Δ	Δ	Δ	Δ	Δ	X	Δ	X
	Starch	Δ	Δ	Δ	Δ	Δ	Δ	Δ	Δ	Δ	X	O	O
60	M_11_	-	Δ	Δ	O	Δ	O	Δ	Δ	Δ	X	Δ	X
	M_21_	-	Δ	Δ	O	Δ	O	Δ	Δ	Δ	X	Δ	X
	M_31_	-	Δ	Δ	O	Δ	O	Δ	Δ	Δ	X	Δ	X
	PANI EB	-	Δ	Δ	Δ	Δ	Δ	Δ	Δ	Δ	X	Δ	X
	Starch	-	Δ	Δ	Δ	Δ	Δ	Δ	Δ	Δ	X	X	O
80	M_11_	-	-	-	O	Δ	-	Δ	Δ	Δ	X	Δ	X
	M_21_	-	-	-	O	Δ	-	Δ	Δ	Δ	X	Δ	X
	M_31_	-	-	-	O	Δ	-	Δ	Δ	Δ	X	Δ	X
	PANI EB	-	-	-	Δ	Δ	-	Δ	Δ	Δ	X	Δ	X
	Starch	-	-	-	Δ	Δ	-	Δ	Δ	Δ	X	X	X

Δ: non-soluble, O: slightly (soluble/dispersion), X: soluble/good dispersion, -: evaporation of the solvents.

**Table 4:** Miller indices and diffraction spacing “d” of starch and PANI-EB.

Product	2θ (°)	hkl	d (Å)
PANI-EB	6.4	(001), (0–11)	13.81
	9.1	(021), (0–21), (003)	9.72
	15.7	(023), (030)	5.64
	19.8	(0–13), (−113)	4.48
	25.4	(1–24), (0–16)	3.51
Starch	5.7	(001)	15.51
	11.4	(111)	7.76
	15.04	(140)	5.89
	17.25	(131)	5.14
	19.59	(103)	4.53
	22.22	(113)	4.00
	24.1	(132)	3.69
	26.58	(142)	3.35

**Table 5:** Degree of crystallinity of starch, PANI-EB, and their biocomposites.

Material	Degree of Crystallinity (%)
Starch	37
M_11_	28
M_21_	25
M_31_	21
PANI EB	19

**Table 6:** Thermal characteristics of PANI, starch, and PANI/starch biocomposites (% weight loss at specific temperatures).

Materials	T 5% (°C)	T 10% (°C)	T 50% (°C)	T 95% (°C)	Residue (%) at 700 °C
PANI EB	50	200	461.3	580.3	0
Starch	50.2	70.2	289.6	-	16.5
M_11_	66.6	206.9	384.6	552.9	0
M_21_	53.8	199.3	518.7	-	29.2
M_31_	56.6	200.1	568.0	-	36.0

**Table 7:** Data obtained from DSC thermograms of starch, PANI, and PANI–starch biocomposites.

Material	1st Peak			2nd Peak		
	Peak (°C)	Onset (°C)	∆H (j/g)	Peak (°C)	Onset (°C)	∆H (j/g)
M_11_	88.19	36.89	254.4	255.65	211.94	121.0
M_21_	84.49	34.28	231.5	233.19	215.19	52.58
				270.08	266.57	1.854
M_31_	68.83	39.02	249.2	237.73	218.07	60.89
PANI EB	94.69	35.33	203.5	234.72	195.81	113.6
Starch	100.01	50.45	439.7	266.36	261.71	9.058
				278.10	273.84	15.82

**Table 8:** Redox parameters from cyclic voltammogram peaks of PANI and PANI/starch biocomposites.

Materials	Scan Rate (mV/s)	Ox	Red
PANI EB	10	84.71	−75.19
	50	352.12	−132.77
	100	506.45	−210.18
M_11_	10	222.24	−587.66
	50	905.14	−879.00
	100	1158.75	−1179.44
M_21_	10	118.56	−146.93
	50	669.08	−399.07
	100	866.21	−548.06
M_31_	10	186.53	−81.03
	50	505.03	−226.46
	100	703.62	−380.19

**Table 9:** ξ_HOMO_, ξ_LUMO_ and related molecular properties of the studied compounds.

Parameter	An	An+	I1	I2	I3	PANI	Amylopectin
ξ_HOMO_ (eV)	−5.105	−5.268	−5.736	−5.736	−5.489	−4.770	−7.056
ξ_LUMO_ (eV)	−1.190	−0.1687	−0.318	−0.318	−0.261	−2.980	0.395
Gap ΔE (eV)	3.916	5.099	5.418	5.418	5.227	1.791	7.450
Electronegativity, χ=−μ	3.147	2.718	3.027	3.027	2.875	3.875	3.331
Hardness, η	1.958	2.550	2.709	2.709	2.614	0.895	3.725
Electrophilicity index, ω	2.530	1.450	1.691	1.692	1.581	8.386	1.489
Fractions of transferred electrons, ΔN	1.608	1.066	1.118	1.118	1.100	4.328	0.894

**Table 10:** The HOMO and LUMO electron density distributions of aniline, polyaniline, and amylopectin.

	An	PANI	Amylopectin
HOMO	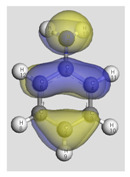	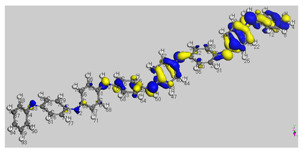	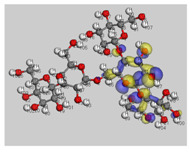
LUMO	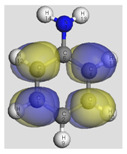	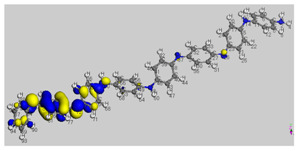	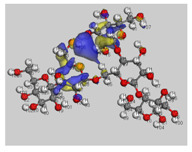

**Table 11:** Fukui analysis of aniline, polyaniline, and amylopectin: The nucleophilic term f_k^+; the electrophilic term f_k^- values of the Fukui function.

	An	PANI	Amylopectin
*f*+	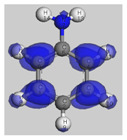	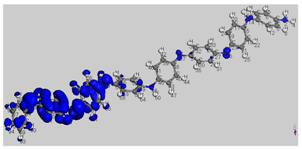	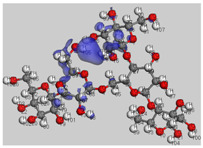
	C(6) = 0.130; (4) = 0.130 C(3) = 0.127; C(1) = 0.127	N(72) 0.076; N(83) 0.074	H(72) 0.107; H(105) 0.194
*f*−	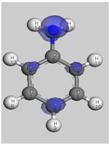	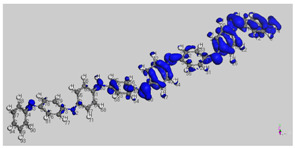	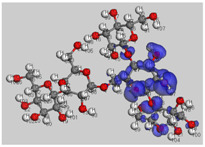
	N(7) 0.193; C(2) 0.110	N(14) 0.037; N(2) 0.029; N(26) 0.027; N(37) 0.030; N(48) 0.031	O(1) 0.105; O(9) 0.115

**Table 13:** The topological characteristics at BCP of interaction contacts.

BCP	Amylopectin	PANI
231	103 (H)	171 (C)
236	26 (O)	174 (H)
239	82 (H)	166 (H)
323	17 (O)	138 (C)
324	75 (H)	136 (C)
361	91 (H)	139 (H)
363	90 (H)	134 (N)

**Table 14:** The topological characteristics at BCP of interaction contacts in the PANI–amylopectin complex.

BCP	ρ(r)	$\nabla^{2}\rho \left(r\right)$	G(r)	V(r)	$E_{HB}\;(\mathrm{eV})$	H = G + V	|V|/G
231	2.32 × 10^−3^	7.35 × 10^−3^	1.34 × 10^−3^	−8.45 × 10^−4^	−4.23 × 10^−4^	4.96 × 10^−4^	0.63
236	3.48 × 10^−3^	1.28 × 10^−2^	2.36 × 10^−3^	−1.52 × 10^−3^	−7.61 × 10^−4^	8.33 × 10^−4^	0.65
239	6.91 × 10^−3^	2.28 × 10^−2^	4.52 × 10^−3^	−3.34 × 10^−3^	−1.67 × 10^−3^	1.18 × 10^−3^	0.74
323	7.83 × 10^−3^	2.72 × 10^−2^	5.42 × 10^−3^	−4.04 × 10^−3^	−2.02 × 10^−3^	1.38 × 10^−3^	0.75
324	5.60 × 10^−3^	2.10 × 10^−2^	4.01 × 10^−3^	−2.76 × 10^−3^	−1.38 × 10^−3^	1.25 × 10^−3^	0.69
361	2.21 × 10^−3^	7.58 × 10^−3^	1.37 × 10^−3^	−8.46 × 10^−4^	−4.23 × 10^−4^	5.24 × 10^−4^	0.62
363	2.32 × 10^−3^	7.35 × 10^−3^	1.34 × 10^−3^	−8.45 × 10^−3^	−4.23 × 10^−4^	4.96 × 10^−4^	0.63

## Abbreviations

(PANI-EB) polyaniline (emeraldine base), (FTIR) Fourier transform infrared, (XRD) X-ray diffraction analysis, (TGA) thermogravimetric analysis, (DTG) Derivative Thermogravimetry, (DSC) Differential scanning calorimetry, (SEM) Scanning Electron Microscopy, (DFT) Density-functional theory, (COSMO-RS) Conductor-like Screening Model for Real Solvents, (AIM methods) Atoms in Molecule, (DMSO) Dimethylsulfoxide, (DMF) Dimethylformamide, (ADF) The Amsterdam Density Functional.
